# Absolute stress myocardial blood flow is reduced in patients with prior cardiac disease despite normal perfusion and ejection fraction on rubidium-82 PET: an Arabian Gulf cohort

**DOI:** 10.3389/fnume.2026.1957482

**Published:** 2026-09-29

**Authors:** Ahmad Alenezi, Masoud Garashi, Layla Ghadenfer, Satish Panchadar, Gautam Biswas, Kawther Ali

**Affiliations:** 1 Department of Radiologic Sciences, Faculty of Allied Health Sciences, Kuwait University, Kuwait City, Kuwait; 2 Nuclear Medicine Department, Kuwait Chest Disease Hospital, Kuwait City, Kuwait; 3 Department of Restorative Sciences, College of Dentistry, Kuwait University, Kuwait City, Kuwait

**Keywords:** Arabian Gulf, cardiac history, myocardial blood flow (MBF), myocardial flow reserve (MFR), myocardial perfusion imaging (MPI), rubidium-82, ventricular function

## Abstract

**Purpose:**

Rubidium-82 (⁸^2^Rb) PET is typically interpreted using perfusion category and left ventricular ejection fraction (LVEF), but absolute myocardial blood flow (MBF) and flow reserve (MFR) may add information. We investigated whether absolute stress MBF differs between patients with and without prior cardiac disease when studies appear normal, in an Arabian Gulf population with high rates of diabetes and obesity. A secondary exploratory aim compared quantitative parameters between predefined PET categories.

**Patients and methods:**

This retrospective single-centre study included 292 source-verified patients (mean age 63.1 ± 12.1 years; 56.8% male) who underwent adenosine-stress ⁸^2^Rb PET/CT, classified by the PET C-category system. The primary analysis compared, within normal-appearing studies (C1 perfusion with resting LVEF ≥ 50%; *n* = 84), those with prior cardiac disease (*n* = 38) and those without (*n* = 46). A secondary exploratory analysis compared these same 46 patients (Normal) with the remaining 246 (Abnormal). Analyses used Mann–Whitney U tests with Cohen's d and 95% confidence intervals, with Bonferroni correction.

**Results:**

Among normal-appearing studies, patients with prior cardiac disease had lower global stress MBF (2.25 vs. 2.99 mL/min/g; d = −0.78, 95% CI −1.22 to −0.33) and lower RCA stress MBF (2.25 vs. 3.29; d = −1.02, −1.47 to −0.56); rest MBF was also lower (d = −0.47, −0.91 to −0.04), whereas MFR (d = −0.37, −0.80 to 0.07), LVEF and LV volumes did not differ. In the secondary comparison, stress MBF and MFR separated the predefined categories comparably (DeLong Z = 0.89, *p* = 0.372) and both more strongly than LVEF (Z = 3.54, *p* < 0.001); because the categories are defined in part by these parameters, this represents relative separation within the classification scheme and not diagnostic accuracy.

**Conclusions:**

In this Arabian Gulf cohort, patients with prior cardiac disease had lower absolute stress MBF despite normal perfusion category and ejection fraction. These exploratory findings support further prospective evaluation of absolute stress MBF as an adjunct to conventional PET interpretation. Because rest and stress flow fell together with a preserved ratio, and because haemodynamic, medication and infarct-related explanations could not be excluded, no mechanism is inferred.

## Introduction

Rubidium-82 (⁸^2^Rb) PET is conventionally interpreted using the categorical perfusion read and the left ventricular ejection fraction (LVEF); however, neither reflects the absolute magnitude of myocardial blood flow, and both may appear normal in the presence of disease. Quantitative PET additionally measures absolute myocardial blood flow (MBF) and myocardial flow reserve (MFR) in physiological units (mL/min/g), providing information on coronary physiology beyond relative perfusion (1). These flow estimates have been validated against nitrogen-13 ammonia (2) and the oxygen-15 water reference technique (3), and current professional-society guidance recommends incorporating MBF and MFR into standard stress PET reporting (4, 5), with recent procedure standards extending quantitative flow assessment to newer tracers (6). This multiparametric framework informs the C1–C7 reporting scheme used locally at our centre, which was developed from the perfusion and flow-reserve criteria in these documents but is not itself defined by them, and, which stratifies studies by perfusion and global MFR, including the C3 category (normal perfusion with reduced MFR) that relative perfusion imaging alone cannot identify (4–6).

Even when perfusion appears normal, impaired flow may persist and carries prognostic significance: a 2025 systematic review and meta-analysis reported that reduced PET-derived MFR independently predicts adverse cardiovascular events (7), and a large multicentre study linked an MFR ≤ 2.0 to increased mortality among patients without perfusion defects (8). Reduced flow in the absence of obstructive epicardial disease defines coronary microvascular dysfunction, a recognised contributor to morbidity, mortality, and heart failure with preserved ejection fraction (9). This phenotype is particularly prevalent in cardiometabolic disease, in which reduced MFR occurs even without epicardial stenosis (10).

Deriving reliable flow estimates from ⁸^2^Rb data depends on the quantification framework. As a generator-produced tracer, ⁸^2^Rb enables accurate cardiac PET without an on-site cyclotron (1) and is now the most widely used PET perfusion agent, with robust diagnostic performance demonstrated in women and higher-body-mass-index patients (11). Reliable absolute quantification nonetheless requires a robust image-processing and kinetic-modelling pipeline (12), and flow-reserve values must be internally validated for reproducibility (13); both were features of the Corridor4DM platform used in this study. Because absolute flow values vary with tracer, scanner, stress agent, kinetic model, and software, as shown in the multi-software RUBY-10 comparison (14), quantitative values and cut-offs are interpretable only within a defined acquisition-and-analysis framework, and protocol standardisation remains an unmet need (1).

Interpretation also depends on the reference population. Flow reference limits are demographically dependent, varying with age and sex such that a single universal threshold is inadequate (15, 16); the same has been shown for 13N-ammonia (17) and 11C-acetate PET (18). Existing ⁸^2^Rb normative data derive almost exclusively from North American and European cohorts, whereas the Arabian Gulf carries a substantially higher burden of diabetes and obesity, Kuwait has among the highest age-standardised diabetes prevalence worldwide (25.6% in 2024) (19). Because diabetes and obesity impair microvascular function and lower baseline flow (9, 10), the entire flow distribution, and the behaviour of absolute flow as a marker of disease, may differ in this population from the cohorts in which it has been characterised.

Whether absolute stress MBF can distinguish patients with prior cardiac disease from those without when both perfusion category and LVEF appear normal has not, to our knowledge, been examined, and never in an Arabian Gulf population. The primary aim of this study was to determine whether, within normal-appearing ⁸^2^Rb PET studies (normal perfusion category and normal resting LVEF), absolute stress MBF differs between patients with and without prior cardiac disease. The secondary, exploratory aim was to compare these quantitative measures between the predefined PET categories, using a consistent Corridor4DM quantification pipeline. Because the categories are defined in part by perfusion and flow reserve, that comparison describes relative separation within the classification scheme rather than diagnostic accuracy.

## Patients and methods

### Study design and patient selection

This retrospective single-centre study is reported in accordance with the STROBE statement for observational studies. It was approved by the institutional ethics committee with a waiver of informed consent for the use of anonymized data (approval no. 2465). The analysis cohort comprised 292 source-verified patients - derived from 330 consecutive studies by de-duplicating repeat scans to the index study and excluding studies without a locatable source report or with internally inconsistent (stress = rest) global flow values - who underwent one-day gated rest-stress ⁸^2^Rb-chloride PET/CT myocardial perfusion imaging (with low-dose CT for attenuation correction) at Kuwait Chest Disease Hospital between January 2018 and December 2023 (Table 1). Rubidium-82 chloride was produced using a CardioGen-82® strontium-82/rubidium-82 generator (Bracco Diagnostics Inc., Princeton, NJ, USA) and delivered intravenously with its dedicated infusion system; pharmacologic stress was induced with adenosine. Patients were classified using the PET reporting C-category scheme. Group definitions for the present analysis are given under Group Definitions.

**Table 1:** Cohort characteristics: analytic cohort, and the primary comparison of prior cardiac disease vs. none within normal-appearing studies.

Characteristic	Analytic cohort (*n* = 292)	Prior cardiac disease (*n* = 38)	No cardiac disease (*n* = 46)	p
Age, median [IQR] (y)	64.0 [54.0–71.0]	62.0 [56.0–69.5]	57.5 [50.0–70.0]	0.225
Female/male	126/166	11/27	26/20	0.021
BMI, median [IQR] (kg/m^2^)	32.0 [27.5–34.9]	31.6 [27.1–35.0]	32.0 [28.5–34.5]	0.776
Obesity (BMI ≥ 30), *n* (%)	182 (62.3)	22 (57.9)	32 (69.6)	0.378
Indication: diagnosis of CAD	82 (28.1)	3 (7.9)	34 (73.9)	—
Indication: revascularisation assessment	151 (51.7)	32 (84.2)	3 (6.5)	—
Indication: risk stratification	25 (8.6)	1 (2.6)	3 (6.5)	—
Indication: pre-operative	30 (10.3)	2 (5.3)	5 (10.9)	—
C-category C1/C2/C3/C4	105/6/47/21	—	—	—
C-category C5/C6/C7	19/32/61	—	—	—
Diabetes, hypertension, dyslipidaemia, smoking	not recorded	not recorded	not recorded	—
Medication use	not recorded	not recorded	not recorded	—
Resting heart rate, blood pressure	not recorded	not recorded	not recorded	—

Normal=C1 perfusion category with resting LVEF ≥ 50% and no cardiac history (*n* = 46); Abnormal=all remaining patients (*n* = 246). The MFR criterion has been removed from this definition to reduce circularity in the secondary comparison. Diabetes, hypertension, dyslipidaemia, smoking, medication use, resting heart rate and blood pressure were not recorded in the imaging database and could not be retrieved. The p column compares Normal vs Abnormal (Mann–Whitney U for continuous variables; chi-square for sex).

Studies were assigned to one of seven local reporting categories combining visual perfusion assessment with global myocardial flow reserve. The distribution was: C1, normal perfusion with MFR ≥ 2.0, 105 studies; C2, abnormal perfusion with MFR < 2.0, 6; C3, normal perfusion with MFR < 2.0, 47; C4, abnormal perfusion with MFR ≥ 2.0, 21; and C5, C6 and C7, abnormal perfusion with abnormal flow reserve in one, two and three vascular territories, 19, 32 and 61 respectively. One study carried an invalid category and was excluded. Indications for the PET study were revascularisation assessment in 151 patients (51.7%), diagnosis of coronary artery disease in 82 (28.1%), pre-operative assessment in 30 (10.3%), risk stratification in 25 (8.6%), and medical board, pre-transplant or transcatheter aortic valve implantation assessment in 4 (1.4%).

Source verification denotes that every quantitative value in the analysis file was checked against the corresponding clinical PET report by the first author, with discrepancies resolved in favour of the source report. This process identified one C1-categorised study whose recomputed global myocardial flow reserve fell below 2.0. Normal resting LVEF was defined as 50% or greater. Perfusion categories were assigned by the reporting nuclear medicine physician at the time of clinical reporting as part of routine care; readings were therefore not blinded to clinical history, were not independently duplicated, and no inter-observer agreement statistic can be computed retrospectively.

This ⁸^2^Rb PET cohort is the subject of companion analyses addressing distinct questions, including sex- and age-stratified reference estimates and the association of flow reserve with all-cause mortality. The present paper is confined to the association between prior cardiac disease and absolute stress flow in normal-appearing studies and does not duplicate those analyses.

### PET C-category classification

Every study was assigned, at the time of clinical reporting, to one of seven categories (C1-C7) that integrate the visual perfusion read, the quantitative global myocardial flow reserve (MFR; abnormal defined as < 2.0, per quantitative PET reporting guidance (4, 5), and - for abnormal-perfusion studies - the number of coronary territories with reduced flow reserve. The categories are: C1, normal perfusion with normal global MFR (≥ 2.0); C2, abnormal perfusion with reduced global MFR (< 2.0); C3, normal perfusion with reduced global MFR (< 2.0); C4, abnormal perfusion with preserved global MFR (≥ 2.0); and C5, C6, and C7, abnormal perfusion with reduced flow reserve in one, two, or three coronary territories, respectively. C1 therefore represents a fully normal study and anchors the Normal reference group, whereas C3 - normal perfusion with reduced reserve - is the pattern that may reflect either microvascular dysfunction or balanced multivessel epicardial disease (Section 4.4). During data verification each study's category was re-derived from the source-report perfusion impression and MFR values; two studies carrying a C3 label had a global MFR ≥ 2.0 and were reclassified out of C3, yielding 45 C3 studies for the microvascular analysis.

### Image acquisition

PET/CT: Rest and stress ⁸^2^Rb PET/CT myocardial perfusion imaging was performed on a dedicated PET/CT scanner. A CT scout was acquired (kV 120, mA 10) followed by a helical CTAC scan (kV 120, mA 145, slice thickness 3.75 mm, pitch 0.531:1, rotation time 0.8 s, CTDIvol 19.85 mGy, DLP 340.75 mGy·cm). Adenosine pharmacologic stress was induced with(0.14 mg/kg/min over 6 min; caffeine and theophylline withheld ≥48 h). ⁸^2^Rb chloride was administered from a CardioGen-82® generator (target approximately 1,110 MBq). Dynamic list-mode data was utilized for 7–8 min with ECG gating during the static phase (8 or 16 frames/cycle) over a single bed position, with a 2-minute pre-scan delay followed by a 4-minute static emission acquisition. OSEM reconstruction method was used(3 iterations, 18 subsets) with time-of-flight and point-spread-function modelling (Q.AC wide-view, VPFX-S; DFOV 70 cm, matrix 128 × 128, voxel size 2.0 mm).

### Group definitions

The Normal group consisted of patients with visually normal stress and rest perfusion, normal global MFR (≥ 2.0), normal resting LVEF, and no history of coronary artery disease, prior myocardial infarction, revascularisation, cardiomyopathy, or congenital heart disease (*n* = 46). All remaining patients constituted the Abnormal group (*n* = 246), spanning abnormal perfusion, impaired flow reserve, reduced LV function, or documented cardiac history. Baseline cardiovascular risk factors (age, sex, BMI, diabetes, hypertension, hyperlipidaemia, family history of CAD, smoking) were systematically recorded.

### Image analysis software

All PET images were processed with Corridor4DM (INVIA, Ann Arbor, MI, USA). The same processing method was used for all datasets.

### Statistical analysis

Analyses were performed in R (RStudio Team, 2026) following APA 7 standards with exact *p*-values and 95% CI for all effect sizes. Normality was assessed by Shapiro–Wilk (non-parametric methods used throughout). Normal-vs.-Abnormal differences used Mann–Whitney U with Cohen's d (95% CI) (20). Discrimination was assessed by ROC analysis [area under the curve (AUC) with 95% CI, and Youden-optimal cut-offs with sensitivity/specificity] and by Mann–Whitney U tests with Cohen's d and 95% confidence intervals. Within the normal-values stratum (C1 + normal LVEF), patients with vs. without prior cardiac history were compared by Mann–Whitney U with Cohen's d. Sex differences used Mann–Whitney U with rank-biserial r; age effects used Kruskal–Wallis H with rank eta-squared. Bonferroni correction was applied across parameter comparisons. To test whether this association was independent of confounders, absolute stress MBF was additionally modelled by multivariable linear regression on prior cardiac history with age, sex, and body mass index as covariates, with documented diabetes added in a sensitivity model. Internal validity of the multivariable model was assessed with a 2,000-replicate Harrell optimism bootstrap: the model was refitted in each bootstrap resample, the difference between its bootstrap-sample and original-sample AUC was averaged to estimate optimism, and this was subtracted from the apparent AUC to yield an optimism-corrected estimate.

## Results

### Cohort characteristics

Of the 292 source-verified patients, 46 met normal criteria and 246 were classified as abnormal (Table 1). The groups were similar in sex distribution (56.5% vs. 40.7% female, *p* = .067) and BMI (31.8 vs. 31.4 kg/m2, *p* = .718); Normal patients were modestly younger (mean 59.1 vs. 63.8 years, *p* = .026). All Shapiro–Wilk tests for the core parameters rejected normality (W < 0.97, *p* < .001), supporting the non-parametric analytical strategy.

### Age effects on LV functional parameters

#### Discrimination of normal from abnormal studies

The largest separation between Normal (*n* = 46) and Abnormal (*n* = 246) patients was greatest in absolute stress flow parameters. Median global stress MBF was 2.99 [IQR 2.36–3.22] mL/min/g in Normal vs. 1.70 [1.31–2.29] in Abnormal (Cohen's d = 1.17), and global MFR was 2.72 [2.26–3.19] vs. 1.87 [1.45–2.33] (d = 0.95); every territorial stress-MBF and MFR difference was highly significant after Bonferroni correction (Table 2, Figure 1). Stress LVEF was higher in Normal patients (median 67 vs. 56%, d = 0.84) and LV volumes were smaller (stress ESV 22 vs. 35 mL). Rest global MBF differed only slightly (1.00 vs. 0.90, d = 0.10, ns).

**Table 2:** Exploratory comparison of quantitative PET parameters between the predefined normal and abnormal categories.

Parameter	Normal (*n* = 46) median	Abnormal (*n* = 246) median	p (MWU)	p (Bonf.)	Cohen's d [95% CI]	Sig.
Stress MBF global, mL/min/g	2.97	1.70	<0.001	<0.001	+1.15 [0.82, 1.48]	***
Stress MBF LAD, mL/min/g	2.70	1.70	<0.001	<0.001	+0.92 [0.60, 1.25]	***
Stress MBF LCX, mL/min/g	2.99	1.68	<0.001	<0.001	+1.15 [0.82, 1.49]	***
Stress MBF RCA, mL/min/g	3.28	1.88	<0.001	<0.001	+1.28 [0.95, 1.62]	***
Rest MBF global, mL/min/g	1.00	0.91	0.021	0.315	+0.03 [−0.28, 0.35]	ns
MFR global (unitless)	2.73	1.86	<0.001	<0.001	+1.00 [0.67, 1.33]	***
MFR LAD (unitless)	2.58	1.80	<0.001	<0.001	+0.76 [0.44, 1.09]	***
MFR LCX (unitless)	2.61	1.80	<0.001	<0.001	+1.13 [0.80, 1.46]	***
MFR RCA (unitless)	3.02	1.95	<0.001	<0.001	+1.10 [0.77, 1.43]	***
Stress LVEF, %	67.0	56.0	<0.001	<0.001	+0.84 [0.51, 1.16]	***
Rest LVEF, %	59.0	55.0	<0.001	0.006	+0.60 [0.28, 0.92]	**
Stress EDV, mL	67.0	81.0	0.007	0.108	−0.45 [−0.77, −0.13]	ns
Stress ESV, mL	22.0	35.0	<0.001	<0.001	−0.60 [−0.92, −0.28]	***

Mann–Whitney U with Bonferroni correction across k = 15 comparisons (*α* = .0033); Cohen's d with pooled SD and 95% CI, positive values indicating higher values in the Normal group; MFR is unitless. *** *p* < 0.001; ** *p* < 0.01.

**Figure 1:**
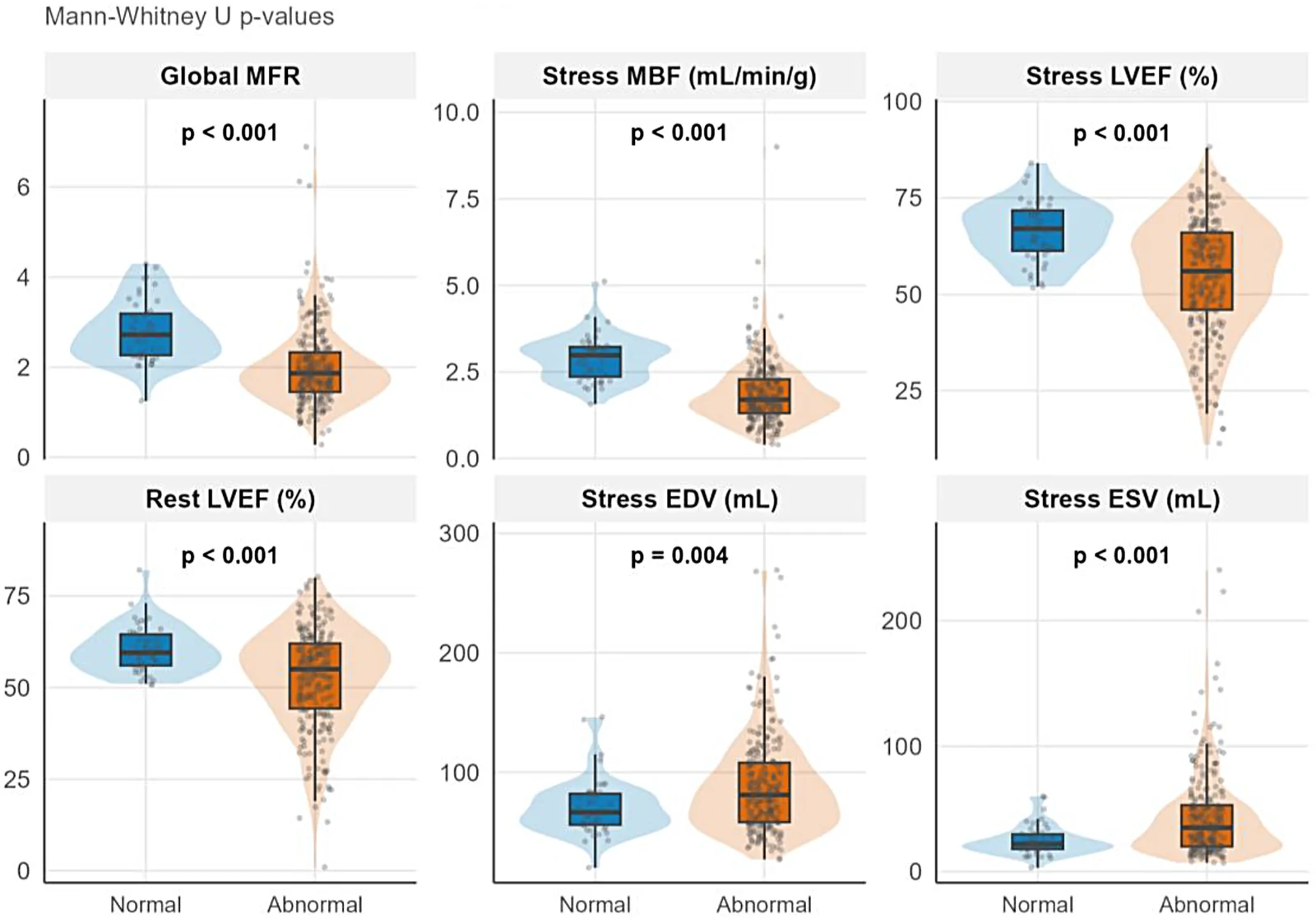
Normal vs. Abnormal for key parameters, with Mann–Whitney U *p*-values on each panel.

On reciever operator cherastristic (ROC) analysis, stress MBF was the strongest single discriminator of an abnormal study. Global stress MBF achieved an AUC of 0.86 (95% CI 0.81–0.90) and territorial stress MBF 0.84–0.86; global MFR reached 0.81 (0.76–0.87), and MFR by territory 0.80–0.81 (Table 3, Figures 2 and 3). LV functional parameters were weaker discriminators (stress LVEF AUC 0.74; stress ESV 0.70). A multivariable model combining global and territorial stress-flow terms achieved an apparent AUC of 0.87 (95% CI 0.83–0.91) at 9.2 events per variable; internal validation by a 2,000-replicate optimism bootstrap showed minimal overfitting (optimism 0.01), giving an optimism-corrected AUC of 0.86. Because this only marginally exceeds global stress MBF alone (AUC 0.86), the multivariable model is not advanced as an independent diagnostic tool.

**Table 3:** Exploratory discrimination of a predefined abnormal PET category.

Parameter	AUC [95% CI]	Cut-off	Sens.	Spec.	Balanced acc.	DeLong vs global stress MBF
Stress MBF global	0.850 [0.802–0.898]	1.99	0.636	0.978	0.807	reference
Stress MBF LAD	0.838 [0.787–0.889]	1.97	0.636	0.956	0.796	Z = 1.42, *p* = 0.156
Stress MBF LCX	0.857 [0.810–0.904]	2.00	0.648	0.956	0.802	Z = −0.69, *p* = 0.490
Stress MBF RCA	0.849 [0.797–0.900]	2.24	0.688	0.911	0.800	Z = 0.12, *p* = 0.902
MFR global	0.827 [0.779–0.875]	2.02	0.632	1.000	0.816	Z = 0.89, *p* = 0.372
MFR LAD	0.816 [0.766–0.866]	2.02	0.615	0.978	0.797	Z = 1.26, *p* = 0.209
MFR LCX	0.823 [0.772–0.875]	1.96	0.599	0.956	0.777	Z = 0.98, *p* = 0.326
MFR RCA	0.822 [0.769–0.875]	2.21	0.652	0.889	0.770	Z = 0.97, *p* = 0.333
Stress LVEF	0.744 [0.679–0.808]	51.50	0.389	1.000	0.694	Z = 3.54, *p* < 0.001

Positive class=abnormal study. AUC confidence intervals and paired comparisons by the DeLong method; cut-offs are Youden-optimal. Balanced accuracy=(sensitivity + specificity)/2; the no-information rate is 0.846, so raw accuracy is not informative and is not reported. Only the comparison against stress LVEF reached significance: no flow parameter discriminated significantly better than any other. Because the categories are defined in part by perfusion and flow reserve, these values describe relative separation within the classification scheme rather than diagnostic accuracy.

**Figure 2:**
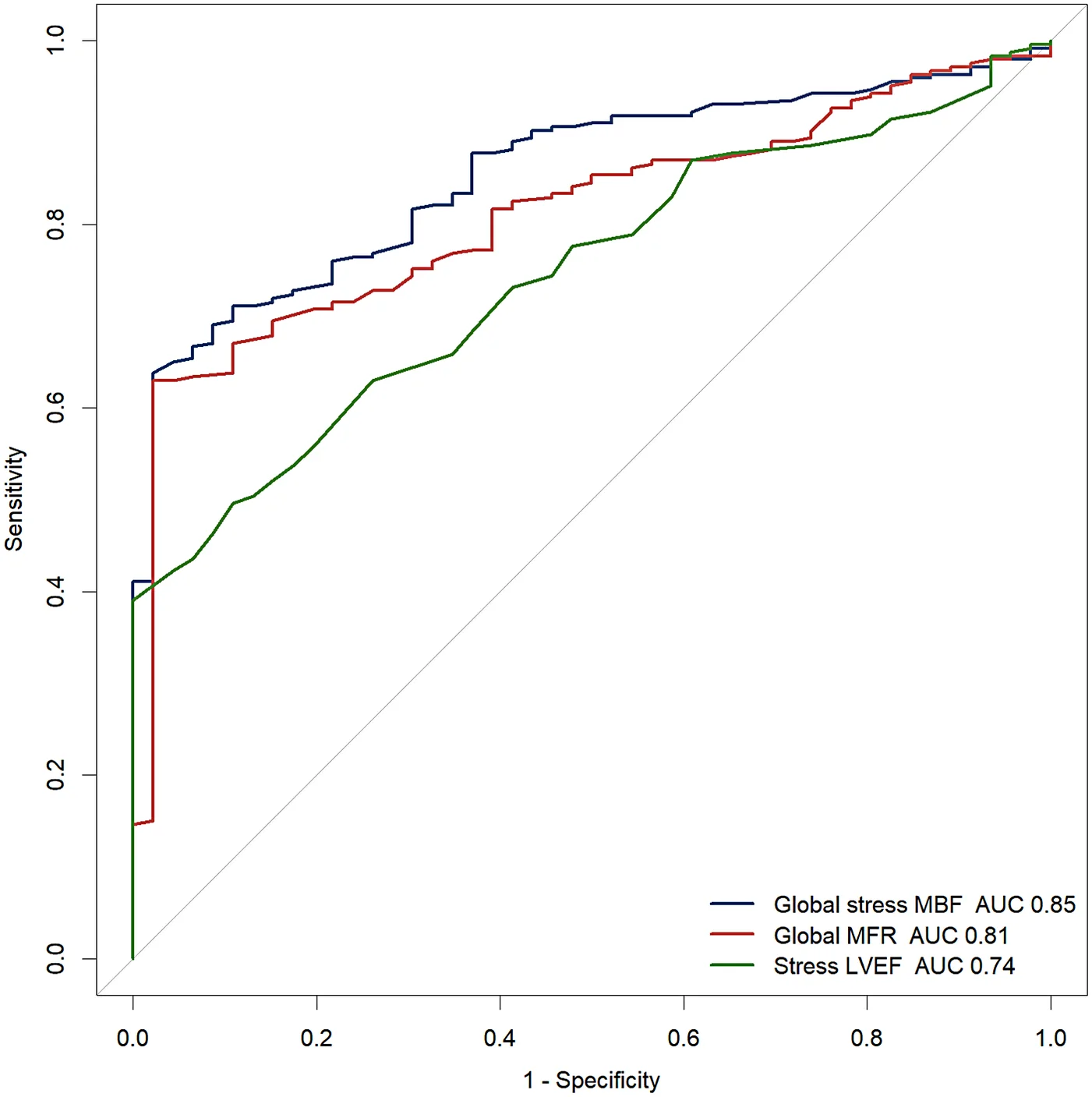
ROC curves for global stress MBF, global MFR and stress LVEF in the exploratory comparison between the predefined normal and abnormal categories. Territorial curves are shown in the Supplementary Material. Because the categories are defined in part by perfusion and flow reserve, these curves describe relative separation within the classification scheme rather than diagnostic accuracy.

**Figure 3:**
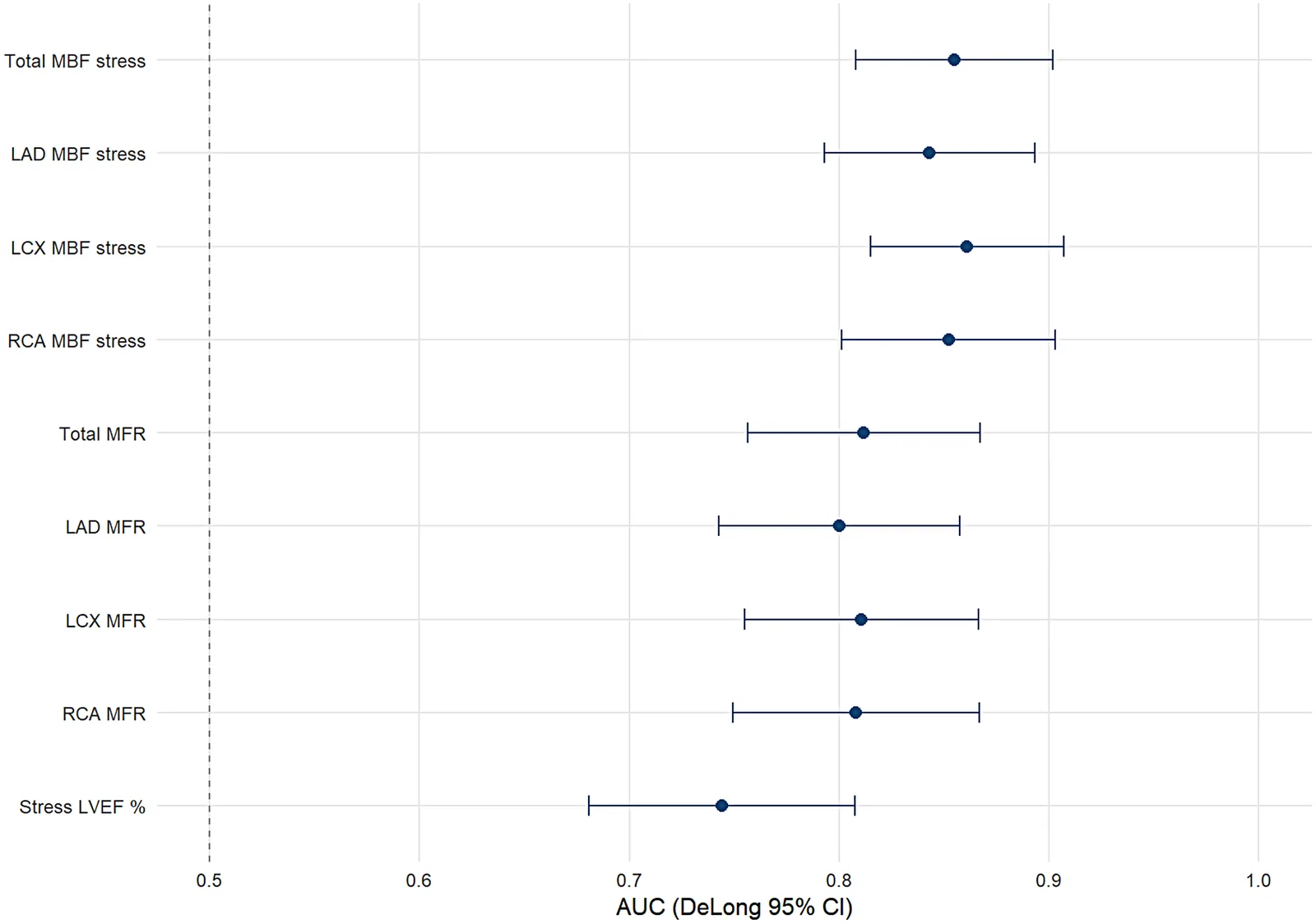
Areas under the curve with deLong 95% confidence intervals for all quantitative parameters. The intervals overlap extensively; paired DeLong testing confirmed no significant difference between any two flow parameters (all *p* ≥ 0.16). The only significant comparison was against stress LVEF (Z = 3.54, *p* < 0.001).

#### Normal-appearing studies: prior cardiac disease vs. none

Within patients with normal-appearing studies (C1 perfusion category with normal resting LVEF, *n* = 84), 38 had documented prior cardiac disease and 46 had none. Despite similar perfusion category and LVEF, patients with prior cardiac history had significantly lower absolute stress MBF (global 2.25 vs. 2.99 mL/min/g, Cohen's d = −0.78, *p* < .001; RCA 2.25 vs. 3.29, d = −1.02, *p* < .001) and lower rest MBF (0.85 vs. 1.00, d = −0.47, *p* = .038, Table 4, Figure 4). Global and territorial MFR trended lower (global MFR 2.43 vs. 2.72, d = −0.37) but were not significant after Bonferroni correction, and LVEF and LV volumes did not differ. Absolute stress flow therefore separates prior-disease patients from truly-normal patients even when the perfusion category and ejection fraction appear normal. This association was independent of confounders; in a multivariable linear model adjusting for age, sex, and BMI, prior cardiac disease remained associated with lower global stress MBF (adjusted *β* = −0.46 mL/min/g, 95% CI −0.81 to −0.12, *p* = .009), an effect that was strongest in the RCA territory (*β* = −0.71, 95% CI −1.11 to −0.32, *p* < .001) and present in each territory (Table 5). Independently associated was observed in older age with lower stress flow, whereas sex and BMI were not, and the association was unchanged by adjustment for documented diabetes (global *β* = −0.47, *p* = .009).

**Table 4:** Primary analysis. normal-appearing studies (C1 perfusion with resting LVEF ≥ 50%): patients with prior cardiac disease (*n* = 38) vs. none (*n* = 46), grouped by parameter family.

Parameter	Prior cardiac disease (*n* = 38) median	No cardiac disease (*n* = 46) median	p (MWU)	p (Bonf.)	Cohen's d [95% CI]	Sig.
Stress MBF global, mL/min/g	2.25	2.99	<0.001	0.001	−0.78 [−1.22, −0.33]	***
Stress MBF LAD, mL/min/g	1.98	2.73	<0.001	0.001	−0.72 [−1.16, −0.27]	***
Stress MBF LCX, mL/min/g	2.13	3.00	<0.001	0.001	−0.71 [−1.15, −0.27]	***
Stress MBF RCA, mL/min/g	2.25	3.29	<0.001	<0.001	−1.02 [−1.47, −0.56]	***
Rest MBF global, mL/min/g	0.85	1.00	0.003	0.036	−0.47 [−0.91, −0.04]	*
MFR global (unitless)	2.43	2.72	0.129	1.000	−0.37 [−0.80, 0.07]	ns
MFR LAD (unitless)	2.34	2.56	0.083	0.996	−0.44 [−0.87, 0.00]	ns
MFR LCX (unitless)	2.47	2.58	0.136	1.000	−0.37 [−0.80, 0.07]	ns
MFR RCA (unitless)	2.46	3.00	0.008	0.100	−0.66 [−1.10, −0.21]	ns
Stress LVEF, %	64.0	67.0	0.381	1.000	−0.21 [−0.64, 0.22]	ns
Rest LVEF, %	58.0	59.5	0.354	1.000	−0.25 [−0.68, 0.18]	ns
Stress EDV, mL	66.5	66.5	0.923	1.000	−0.06 [−0.49, 0.37]	ns
Stress ESV, mL	23.5	22.0	0.877	1.000	+0.04 [−0.39, 0.47]	ns

Mann–Whitney U with Bonferroni correction and Cohen's d. *** *p* < 0.001; ** *p* < 0.01.

**Figure 4:**
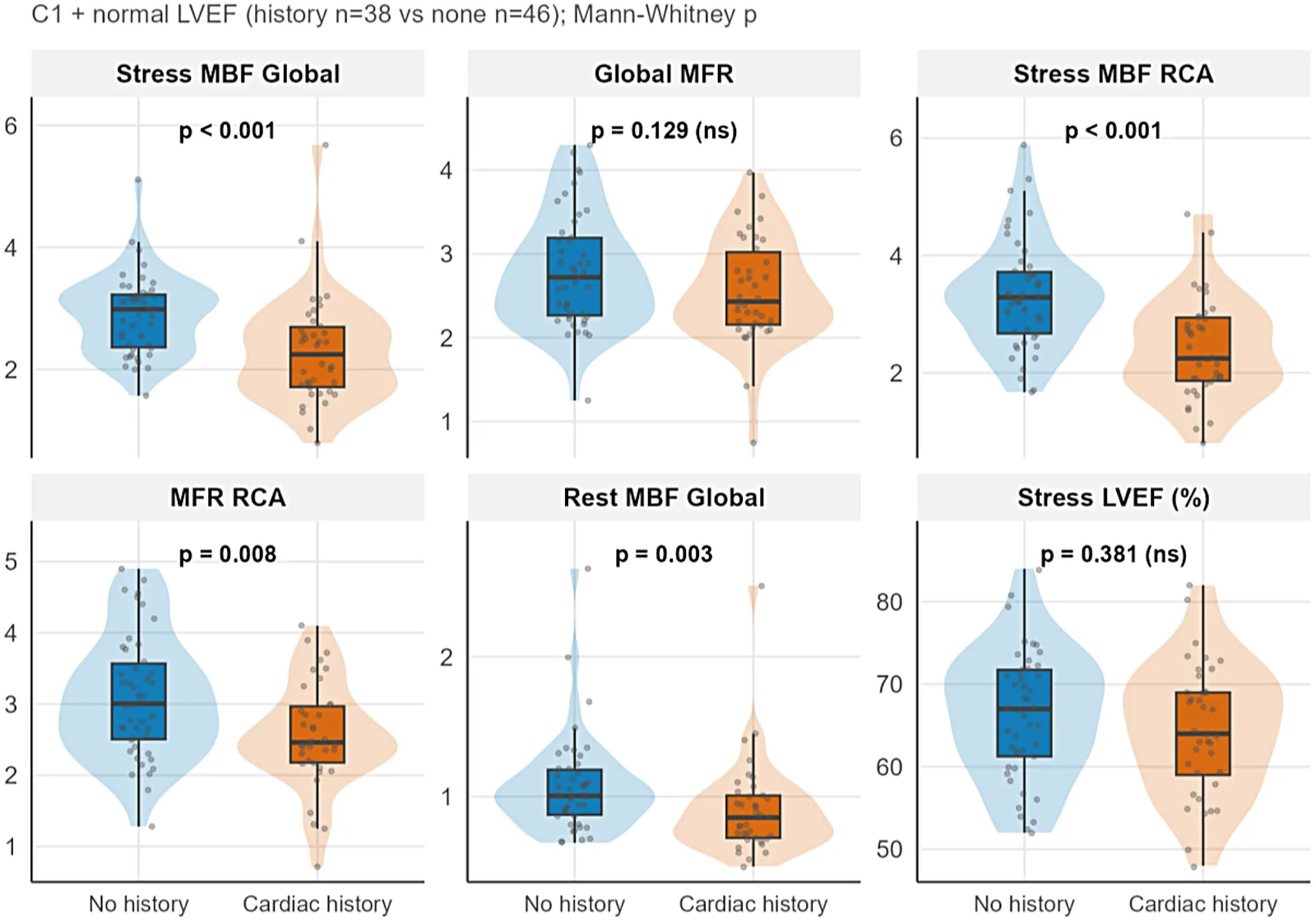
Normal-appearing studies (C1 perfusion with resting LVEF ≥ 50%): prior cardiac disease (*n* = 38) vs. none (*n* = 46). Key parameters with Mann–Whitney U *p*-values and Cohen's d with 95% confidence intervals.

**Table 5:** Multivariable linear regression of absolute stress MBF on prior cardiac disease within the normal-appearing subgroup (*n* = 84), adjusted for age, sex and body mass index.

Territory	Predictor^a^	*β*	95% CI	p
Global	Prior cardiac disease	−0.462	−0.790 to −0.099^a^	0.009
	Age (per year)	−0.021	−0.035 to −0.007	0.003
	Male sex	−0.285	−0.650 to 0.080	0.124
	BMI (per kg/m^2^)	−0.018	−0.047 to 0.012	0.240
LAD	Prior cardiac disease	−0.510	−0.862 to −0.136^a^	0.008
	Age (per year)	−0.016	−0.032 to −0.001	0.038
	Male sex	−0.113	−0.511 to 0.285	0.574
	BMI (per kg/m^2^)	−0.013	−0.046 to 0.019	0.415
LCX	Prior cardiac disease	−0.402	−0.755 to 0.009^a^	0.032
	Age (per year)	−0.022	−0.037 to −0.007	0.005
	Male sex	−0.450	−0.838 to −0.062	0.024
	BMI (per kg/m^2^)	−0.017	−0.049 to 0.015	0.285
RCA	Prior cardiac disease	−0.714	−1.112 to −0.317	0.0006
	Age (per year)	−0.025	−0.041 to −0.008	0.004
	Male sex	−0.505	−0.927 to −0.083	0.020
	BMI (per kg/m^2^)	−0.026	−0.061 to 0.008	0.130

a Adjusted R^2^ = 0.194 (global), 0.126 (LAD), 0.184 (LCX), 0.281 (RCA); residual standard error 0.740–0.855; all variance inflation factors ≤ 1.27. Homoscedasticity satisfied in every model (Breusch–Pagan *p* = 0.10–0.36). ^1^Residuals departed from normality in the global, LAD and LCX models (Shapiro–Wilk *p* < .05), so bootstrap percentile 95% CIs (2,000 resamples) are reported for the exposure coefficient in those models; the LCX interval crosses zero. RCA residuals were normal (*p* = .53) and its interval is model-based. Diagnostic plots: Supplementary Figure S1.

Among the 84 normal-appearing studies, patients with prior cardiac disease had lower absolute stress myocardial blood flow than those without, globally (median 2.25 vs. 2.99 mL/min/g; d = −0.78, 95% CI −1.22 to −0.33) and in every territory, with the largest deficit in the RCA distribution (2.25 vs. 3.29; d = −1.02, −1.47 to −0.56). Rest MBF was also lower (0.85 vs. 1.00; d = −0.47, −0.91 to −0.04). Global MFR was numerically lower but did not differ significantly (2.43 vs. 2.72; d = −0.37, −0.80 to 0.07), and neither ejection fraction nor left ventricular volumes differed between the groups. The 46 patients without cardiac history in this primary analysis are the same 46 patients who constitute the Normal group in the secondary comparison.

In multivariable models adjusted for age, sex and body mass index, prior cardiac disease remained independently associated with lower absolute stress MBF globally (*β* = −0.462, bootstrap 95% CI −0.790 to −0.099; *p* = .009) and in the LAD (*β* = −0.510, −0.862 to −0.136) and RCA (*β* = −0.714, −1.112 to −0.317) territories; the LCX association was weaker and its bootstrap interval crossed zero (*β* = −0.402, −0.755 to 0.009). Age was independently associated with lower stress flow in every territory. Referral indication could not be included as a covariate because it was almost entirely collinear with the exposure.

## Discussion

### Summary and contributions

This study of an Arabian Gulf cohort found that, among studies that appeared normal by perfusion category and ejection fraction, patients with prior cardiac disease had lower absolute stress and rest myocardial blood flow. In an exploratory secondary comparison, absolute stress MBF and MFR separated the predefined PET categories comparably (DeLong Z = 0.89, *p* = 0.372), and both more strongly than ejection fraction (Z = 3.54, *p* < 0.001); no flow parameter discriminated significantly better than another. This is discussed further below for ^82^Rb PET for this population. A multivariable flow model produced a concordant but exploratory apparent AUC of 0.87 that corrected to 0.86 on internal bootstrap validation; because this only marginally exceeds global stress MBF alone, it is not advanced as an independent diagnostic tool. The cohort comprised consecutive Kuwaiti patients with elevated metabolic risk (average BMI 31.5 kg/m2), a group underrepresented in previous PET studies (15).

This analysis offers three additional strengths. First, MBF, MFR, and LV function are reported together from a single integrated cohort and analysis system, enabling direct comparison of their discriminatory capacity within the same patients. Second, the study utilizes a de-duplicated, source-verified cohort in which each patient was matched to an original PET report. Third, the analysis clearly distinguishes confirmed flow findings from exploratory results, designating the latter as directions for future research rather than definitive conclusions.

### MBF and MFR: clinical context

Absolute stress MBF and global MFR were the strongest discriminators of an abnormal study, with global and territorial stress MBF reaching areas under the curve of 0.84–0.86 and global MFR 0.81. Because the Normal group was defined in part by normal perfusion and global MFR >= 2, discrimination by these specific parameters is partly definitional, and the interpretable signal is the magnitude of separation rather than any ranking among the flow parameters, since paired DeLong testing showed no significant difference between them (global stress MBF vs. global MFR, Z = 0.89, *p* = 0.372). Absolute stress MBF, which is not itself a defining criterion, separated the groups with a Cohen's d of 1.15 (95% CI 0.82 to 1.48). A further observation strengthens the physiological interpretation: among patients whose perfusion category and LVEF appeared normal, those with prior cardiac disease had significantly lower absolute stress and rest MBF than truly-normal patients (global stress MBF 2.25 vs. 2.99 mL/min/g, d = −0.78), even though MFR and LVEF did not separate them. Quantitative absolute flow therefore detects disease that categorical perfusion reading and ejection fraction miss - concordant with recent evidence that reduced MFR independently predicts death and myocardial infarction regardless of diabetes status and incremental to calcium score and perfusion (21). The prognostic relevance of impaired quantitative flow despite preserved perfusion is well established: reduced coronary flow capacity predicts adverse events even when semiquantitative perfusion reads as normal (22), and quantitative PET perfusion adds independent risk stratification in patients with prior coronary artery bypass grafting (23).

The highest reserve was observed in the RCA territory, consistent with regional variation reported in prior (⁸^2^Rb) PET data, and supports territory-specific assessment alongside global MFR, since regional deficits may be obscured by global averaging. Typically, absolute flow metrics are sensitive to kinetic model and software platform (14, 24), so cut-offs and quantitative values should be interpreted within the pipeline that produced them.

The pattern observed merits careful interpretation. Stress MBF and rest MBF both fell in the prior-cardiac-disease group (d = −0.78 and −0.47) while the ratio was preserved (MFR d = −0.37, not significant). A proportional reduction in rest and stress flow with a preserved ratio is not the characteristic signature of microvascular dysfunction; it is what would be expected from a lower rate-pressure product or from reduced viable myocardial mass. Because resting heart rate and blood pressure were not recorded, we cannot exclude a haemodynamic explanation; because medication use was not recorded, we cannot exclude an effect of beta-blockade or nitrates; and because prior infarction cannot be identified within the composite exposure, we cannot exclude reduced viable myocardium or partial-volume effects from transmural scar. We therefore report this as an association and infer no mechanism.

The RCA territory showed both the highest flow reserve in the cohort overall and the largest deficit in the prior-cardiac-disease group. Inferior-wall attenuation-correction misregistration and prior inferior infarction are both plausible contributors that these data cannot exclude, and the two RCA observations should be read together rather than as independent findings.

A further consideration bears directly on interpretation. Referral indication was almost entirely collinear with the exposure: 84% of the prior-cardiac-disease group were referred for revascularisation assessment, against 7% of those without. Because indication could not be adjusted for, and because medication status was not recorded, we cannot separate a disease-related reduction in flow from a pharmacological one. This is the leading alternative explanation for the primary finding and it cannot be excluded with the available data.

We are not in a position to specify how a reporting physician should act on a given absolute flow value. Establishing that would require prospective ascertainment of the constituent diagnoses within the cardiac-history composite, haemodynamic and medication data, and outcome follow-up, none of which was available here.

### The C3 pattern against angiography

The C3 category - normal perfusion with reduced global MFR - is the pattern of greatest mechanistic interest, because it can reflect either genuine microvascular dysfunction (angiographically clean epicardial arteries) (25) or balanced multivessel epicardial disease that lowers flow globally without producing a regional perfusion defect (26); the two are indistinguishable on PET alone. In our cohort 45 patients had the C3 pattern, but only 4 (9%) underwent coronary angiography, and all four showed obstructive epicardial disease rather than clean arteries (Supplementary Table S1). The two with an angiogram within the imaging window (+12 and +45 days) both had balanced triple-vessel disease, directly illustrating how balanced macrovascular disease can masquerade as C3. This should not be over-interpreted: patients were referred to angiography selectively - typically for angina, prior coronary disease, or an acute coronary event - so this small subset is strongly enriched for obstructive disease (verification bias), indeed, across the whole cohort 68 patients underwent catheterisation and virtually none had angiographically normal coronary arteries, confirming that angiography here was reserved almost exclusively for patients with a high pre-test probability of obstructive disease; while the 41 C3 patients without angiography, presumably of lower clinical suspicion, may well include genuine microvascular dysfunction that remained invisible to a purely non-invasive dataset. The nature of C3 in this population therefore cannot be resolved with the available data. Systematically distinguishing microvascular from balanced macrovascular C3 - the question this pattern poses - requires prospective, protocol-driven angiography and is an important direction for future work. This dual mechanism is recognised in the literature - dedicated PET reporting frameworks now flag microvascular dysfunction when perfusion is normal but quantitative flow is reduced (25), while quantitative water-PET studies show that extensive but balanced reductions in myocardial blood flow are a hallmark of multivessel obstructive disease (26) - underscoring that the C3 pattern cannot be assigned a mechanism without invasive correlation.

Echocardiography, ordered far more widely than angiography and less selectively, offers a partial resolution that catheterisation alone cannot. Because genuine microvascular dysfunction preserves regional systolic function whereas prior epicardial infarction produces a regional wall-motion abnormality, the resting echocardiogram provides an independent readout of the macrovascular substrate. A resting echocardiogram was available in 24 of the 45 C3 patients (53%), against only 4 (9%) with angiography. Among the 24, seven (29%) showed a regional wall-motion abnormality indicating prior infarction or macrovascular territory disease - including all three who also had angiography, in whom the abnormal territory matched the culprit vessel (inferior hypokinesis with right-coronary disease) - whereas ten (42%) had explicitly preserved regional function despite the reduced flow reserve, the pattern expected in true microvascular dysfunction; the remaining seven did not comment on regional wall motion (Supplementary Table S2). Thus, while angiography identified only the obstructive tail, the addition of echocardiography indicates that C3 is genuinely mixed, comprising both a macrovascular-masquerade subgroup and a distinct microvascular-pattern subgroup. This remains exploratory: echocardiography detects established wall-motion abnormality rather than active ischaemia and so under-identifies balanced disease without prior infarction, *post-hoc* and post-revascularisation studies were kept separate, and 23 C3 patients had no echocardiogram.

Only 4 of 47 C3 patients underwent angiography, of which two were evaluable, and all four had obstructive epicardial disease. Because patients with angiographically unobstructed arteries are unlikely to be referred for catheterisation, this verified subset is subject to near-total verification bias and cannot establish the prevalence of either phenotype. These data cannot distinguish microvascular dysfunction from balanced epicardial disease, and no mechanism is inferred.

### Limitation

This study has several limitations. The exposure was a composite record-derived cardiac history; its constituent diagnoses, prior infarction, revascularisation, cardiomyopathy and congenital disease,were not recorded separately, so the composite is too heterogeneous to support mechanistic inference and a sensitivity analysis excluding non-ischaemic causes was not possible. The timing of the qualifying event, and whether revascularised patients were studied before or after intervention, were likewise unrecorded; this matters particularly because revascularisation assessment was the commonest referral indication (51.7%). Cardiovascular risk-factor data other than body mass index, medication use, resting heart rate and blood pressure, renal function and semi-quantitative perfusion scores were not recorded in the imaging database and could not be retrieved, so rate-pressure-product normalisation and adjustment for these factors were not possible. Perfusion categories were assigned during routine clinical reporting and were therefore neither blinded nor independently duplicated, and no inter-observer agreement could be computed. The Normal group comprised clinically referred patients and was defined in part by the PET read itself, so the secondary comparison describes separation within the study's own classification scheme and not diagnostic accuracy for obstructive disease. The primary subgroups are small (*n* = 38 and 46), and the confidence intervals reported alongside each effect size make the resulting imprecision explicit. Finally, no outcome data are reported, so whether reduced absolute stress flow predicts clinical events cannot be addressed here.

Clinical indication was almost perfectly confounded with the exposure: 32 of the 38 patients with prior cardiac disease were referred for revascularisation assessment, whereas 34 of the 46 without were referred for diagnosis of coronary artery disease. Indication could not therefore be included as a covariate. This matters because patients undergoing revascularisation assessment are more likely to be receiving beta-blockers and nitrates, both of which reduce measured myocardial blood flow, and medication data were not available. An appreciable part of the observed flow difference may therefore be pharmacological rather than disease-related, and the two cannot be separated in these data.

Residuals departed from normality in three of the four regression models; bootstrap confidence intervals are therefore reported for the exposure coefficient in those models. The LCX interval crosses zero, so that territorial association should be regarded as weaker than the global, LAD and RCA findings.

## Conclusions

In this Arabian Gulf cohort, patients with prior cardiac disease had lower absolute stress and rest myocardial blood flow despite a normal perfusion category and preserved ejection fraction. In an exploratory secondary comparison, absolute stress MBF and MFR separated the predefined PET categories comparably, and both more strongly than ejection fraction; because the categories are defined in part by these parameters, that separation is partly definitional and does not represent diagnostic accuracy. Because rest and stress flow fell together with a preserved ratio, and because haemodynamic, medication and infarct-related explanations could not be excluded, no mechanism is inferred. These findings are hypothesis-generating and support further prospective evaluation of absolute stress MBF as an adjunct to conventional PET interpretation, consistent with existing society guidance on quantitative reporting. Because the analysis was cross-sectional and disease status was defined by documented history rather than incident events, prospective outcome-linked studies are required to establish whether reduced absolute stress MBF predicts clinical events.

## Data Availability

The raw data supporting the conclusions of this article will be made available by the authors upon reasonable request.

## Supplementary material

SUPPLEMENTARY TABLE S1

C3 patients (normal perfusion + reduced global MFR) who underwent coronary angiography (4 of 47). All showed obstructive epicardial disease; none had angiographically clean arteries. Two cases were excluded from interpretation (interval revascularisation; angiogram preceding the PET by 257 days), leaving two evaluable studies.

SUPPLEMENTARY TABLE S2

Echocardiographic regional wall-motion assessment of the C3 pattern (normal perfusion + reduced global MFR). Resting echocardiogram available in 24 of 47 C3 patients; 23 had no echocardiogram.

SUPPLEMENTARY FIGURE S1

Residual diagnostics for the multivariable model of global stress myocardial blood flow (*n* = 84). Panels show residuals versus fitted values, a normal quantile–quantile plot, scale–location, and residuals versus leverage. The quantile–quantile plot demonstrates the departure from normality (Shapiro–Wilk *p* < .001) that prompted the use of bootstrap confidence intervals for the exposure coefficient; residuals versus fitted values show no systematic pattern, consistent with the non-significant Breusch–Pagan test (*p* = 0.341).
