# Supplementary material for: Absolute stress myocardial blood flow is reduced in patients with prior cardiac disease despite normal perfusion and ejection fraction on rubidium-82 PET: an Arabian Gulf cohort
Source: Front Nucl Med. 2026 Sep 29;6:1957482. doi: 10.3389/fnume.2026.1957482 (PMC13658550; doi:10.3389/fnume.2026.1957482)
Supplement: Supplementary file 1 [file Datasheet1.pdf]

STROBE Statement—checklist of items that should be included in reports of observational studies

*Revised title: Absolute stress myocardial blood flow is reduced in patients with prior cardiac disease despite normal perfusion and ejection fraction on Rubidium-82 PET: an Arabian Gulf cohort*

(previously: "Beyond normal perfusion and ejection fraction: absolute stress myocardial blood flow reflects prior cardiac disease on Rubidium-82 PET in an Arabian Gulf cohort")

| | Item No | Recommendation | Line no. |
|---|---|---|---|
| **Title and abstract** | 1 | (*a*) Indicate the study's design with a commonly used term in the title or the abstract | Line 1 (title); lines 30–34 (abstract) |
| | | (*b*) Provide in the abstract an informative and balanced summary of what was done and what was found | Lines 30–61 |
| **Introduction** | | | |
| Background/rationale | 2 | Explain the scientific background and rationale for the investigation being reported | Lines 66–120 |
| Objectives | 3 | State specific objectives, including any prespecified hypotheses | Lines 115–128 |
| **Methods** | | | |
| Study design | 4 | Present key elements of study design early in the paper | Lines 130–144 |
| Setting | 5 | Describe the setting, locations, and relevant dates, including periods of recruitment, exposure, follow-up, and data collection | Lines 130–137 |
| Participants | 6 | (*a*) *Cohort study*—Give the eligibility criteria, and the sources and methods of selection of participants. Describe methods of follow-up<br>*Case-control study*—Give the eligibility criteria, and the sources and methods of case ascertainment and control selection. Give the rationale for the choice of cases and controls<br>*Cross-sectional study*—Give the eligibility criteria, and the sources and methods of selection of participants | Lines 138–161 |
| | | (*b*) *Cohort study*—For matched studies, give matching criteria and number of exposed and unexposed<br>*Case-control study*—For matched studies, give matching criteria and the number of controls per case | Not applicable (unmatched) |
| Variables | 7 | Clearly define all outcomes, exposures, predictors, potential confounders, and effect modifiers. Give diagnostic criteria, if applicable | Lines 145–161; Table 1 (line 166) |
| Data sources/ measurement | 8* | For each variable of interest, give sources of data and details of methods of assessment (measurement). Describe comparability of assessment methods if there is more than one group | Lines 154–161 |
| Bias | 9 | Describe any efforts to address potential sources of bias | Lines 154–161 (unblinded reporting); lines 441–471 |

| | | | |
|---|---|---|---|
| | | | (bias) |
| Study size | 10 | Explain how the study size was arrived at | Lines 138–153; Figure 1 (flow diagram) |
| Quantitative variables | 11 | Explain how quantitative variables were handled in the analyses. If applicable, describe which groupings were chosen and why | Lines 145–153; Methods, statistical analysis |
| Statistical methods | 12 | (*a*) Describe all statistical methods, including those used to control for confounding | Methods, statistical analysis; Table 5 (line 316) |
| | | (*b*) Describe any methods used to examine subgroups and interactions | Lines 291–306; Tables 3–4 (lines 265, 307) |
| | | (*c*) Explain how missing data were addressed | Methods, statistical analysis (complete-case analysis; no imputation) |
| | | (*d*) *Cohort study*—If applicable, explain how loss to follow-up was addressed<br>*Case-control study*—If applicable, explain how matching of cases and controls was addressed<br>*Cross-sectional study*—If applicable, describe analytical methods taking account of sampling strategy | Not applicable (cross-sectional; no follow-up) |
| | | (*e*) Describe any sensitivity analyses | Lines 435–439 (C3 subgroup); lines 467–471 (bootstrap CIs) |

Continued on next page

| | | | |
|---|---|---|---|
| **Results** | | | |
| Participants | 13* | (a) Report numbers of individuals at each stage of study—eg numbers potentially eligible, examined for eligibility, confirmed eligible, included in the study, completing follow-up, and analysed | Lines 138–153; Figure 1 (flow diagram) |
| | | (b) Give reasons for non-participation at each stage | Lines 138–144 (exclusions and reasons) |
| | | (c) Consider use of a flow diagram | Figure 1 (flow diagram) |
| Descriptive data | 14* | (a) Give characteristics of study participants (eg demographic, clinical, social) and information on exposures and potential confounders | Table 1 (line 166); lines 145–153 |
| | | (b) Indicate number of participants with missing data for each variable of interest | Table 1 (line 166) footnote; lines 441–458 |
| | | (c) *Cohort study*—Summarise follow-up time (eg, average and total amount) | Not applicable (cross-sectional; no follow-up) |
| Outcome data | 15* | *Cohort study*—Report numbers of outcome events or summary measures over time | Not applicable |
| | | *Case-control study*—Report numbers in each exposure category, or summary measures of exposure | Not applicable |
| | | *Cross-sectional study*—Report numbers of outcome events or summary measures | Lines 291–299; Tables 2 and 4 (lines 247, 307) |
| Main results | 16 | (*a*) Give unadjusted estimates and, if applicable, confounder-adjusted estimates and their precision (eg, 95% confidence interval). Make clear which confounders were adjusted for and why they were included | Lines 291–306; Tables 4 and 5 (lines 308, 315) |
| | | (*b*) Report category boundaries when continuous variables were categorized | Lines 145–153 (C-category definitions); line 157 (LVEF ≥ 50%) |
| | | (*c*) If relevant, consider translating estimates of relative risk into absolute risk for a meaningful time period | Not applicable (no relative-risk estimates) |
| Other analyses | 17 | Report other analyses done—eg analyses of subgroups and interactions, and sensitivity analyses | Lines 300–306; lines 435–439; Table 5 (line 316) |
| **Discussion** | | | |
| Key results | 18 | Summarise key results with reference to study objectives | Lines 326–331; lines 472–480 |
| Limitations | 19 | Discuss limitations of the study, taking into account sources of potential bias or imprecision. Discuss both direction and magnitude of any potential bias | Lines 441–471 |
| Interpretation | 20 | Give a cautious overall interpretation of results considering objectives, limitations, multiplicity of analyses, results from similar studies, and other relevant evidence | Lines 371–393; lines 472–480 |
| Generalisability | 21 | Discuss the generalisability (external validity) of the study results | Lines 441–458; lines 383–393 |
| **Other information** | | | |

| | | | |
|---|---|---|---|
| Funding | 22 | Give the source of funding and the role of the funders for the present study and, if applicable, for the original study on which the present article is based | Front matter (source of funding: none) |

*Give information separately for cases and controls in case-control studies and, if applicable, for exposed and unexposed groups in cohort and cross-sectional studies.

**Note:** An Explanation and Elaboration article discusses each checklist item and gives methodological background and published examples of transparent reporting. The STROBE checklist is best used in conjunction with this article (freely available on the Web sites of PLoS Medicine at http://www.plosmedicine.org/, Annals of Internal Medicine at http://www.annals.org/, and Epidemiology at http://www.epidem.com/). Information on the STROBE Initiative is available at www.strobe-statement.org.
